# Identification of Peptides in Flowers of *Sambucus nigra* with Antimicrobial Activity against Aquaculture Pathogens

**DOI:** 10.3390/molecules23051033

**Published:** 2018-04-27

**Authors:** Claudio Andrés Álvarez, Andrés Barriga, Fernando Albericio, María Soledad Romero, Fanny Guzmán

**Affiliations:** 1 Laboratorio de Fisiología y Genética Marina (FIGEMA), Centro de Estudios Avanzados en Zonas Áridas (CEAZA), Coquimbo 1781421, Chile; claudio.alvarez@ceaza.cl; 2 Facultad de Ciencias del Mar, Universidad Católica del Norte, Coquimbo 1781421, Chile; msromero@ucn.cl; 3 Centro AquaPacífico, Coquimbo 1781421, Chile; 4 Unidad de Espectrometría de Masas, Facultad de Ciencias Químicas y Farmacéuticas, Universidad de Chile, Santiago 8380494, Chile; anbarr@ciq.uchile.cl; 5 Department of Organic Chemistry and CIBER-BBN, Networking Centre on Bioengineering, Biomaterials and Nanomedicine, University of Barcelona, Barcelona 08007, Spain; albericio@ukzn.ac.za; 6 School of Chemistry, University of KwaZulu-Natal, Durban 4001, South Africa; 7 Núcleo de Biotecnología de Curauma, Pontificia Universidad Católica de Valparaíso, Valparaíso 2373223, Chile

**Keywords:** *S. nigra*, peptides, antimicrobial activity, fish pathogens, membrane damage

## Abstract

The elder (*Sambucus* spp.) tree has a number of uses in traditional medicine. Previous studies have demonstrated the antimicrobial properties of elderberry liquid extract against human pathogenic bacteria and also influenza viruses. These properties have been mainly attributed to phenolic compounds. However, other plant defense molecules, such as antimicrobial peptides (AMPs), may be present. Here, we studied peptide extracts from flowers of *Sambucus nigra* L. The mass spectrometry analyses determined peptides of 3 to 3.6 kDa, among them, cysteine-rich peptides were identified with antimicrobial activity against various Gram-negative bacteria, including recurrent pathogens of Chilean aquaculture. In addition, membrane blebbing on the bacterial surface after exposure to the cyclotide was visualized by SEM microscopy and SYTOX Green permeabilization assay showed the ability to disrupt the bacterial membrane. We postulate that these peptides exert their action by destroying the bacterial membrane.

## 1. Introduction

Herbal medicine accounts for a considerable share of the pharmaceutical market. In this regard, the global market value of medicinal plant products exceeds US$7 billion a year [1,2]. One medicinal plant is the elder tree, whose berries are widely used in herbal medicine. Previous studies have demonstrated that these berries contain various compounds with high biological activity, such as flavonoids, proanthocyanidins, anthocyanins, and phenolic acids [3,4]. These compounds confer protection against oxidative stress that is caused by reactive oxygen species, which are known to be involved in disorders, like cancer and hypertension [5]. Moreover, elderberry liquid extract shows activity against human pathogenic bacteria and also influenza viruses [6]. 

In addition to these compounds, antimicrobial peptides (AMPs) perform a key role in plant defense against pathogens [7]. Plant AMPs are generally rich in cysteine residues that form multiple disulfides. Cysteine-rich peptides (CRP) families include thionins, defensins, hevein-like peptides, cyclotides, lipid transfer proteins, and the α-hairpinin and snakins family [8,9,10]. In this regard, studies have addressed the use of plant AMPs as substitutes of chemical preservatives and insecticides in agriculture applications [11,12]. In addition, the features of these antimicrobial molecules make them suitable for use in animal farming and public health, thereby decreasing the use of antibiotics. 

Plant AMPs have been isolated and characterized in roots, leaves, and seeds, although they are also present in flower tissues [13]. AMPs from flowers have been characterized on the basis of cationic charge, thereby pointing to lipid bilayer permeabilization as a possible action mechanism [13]. However, given that flowers have received less attention than other tissues, they may hold a number of as yet undiscovered AMPs with biotechnological applications.

Herbal medicine is a long-standing tradition in the Mapuche communities of southern Argentina and Chile [14]. Among the plants that are used for this purpose is *Sambucus nigra* L. (common name “Sauco” in Spanish), which bears berries that contain anti-oxidant phytonutrients that are similar to those found in other elderberries [15]. However, the antimicrobial properties of this medicinal plant have not been established to date. Here, we developed an experimental procedure to obtain a peptide extract from *S. nigra* flowers. Cysteine-rich peptides (CRPs) were identified by mass spectrometry, and the peptide extracts were analyzed for antimicrobial properties, including activity against pathogen bacteria that affect Chilean aquaculture. In addition, scanning electron microscopy (SEM) assays were performed in order to directly observe the response of bacterial cell morphology and membrane integrity to the treatment with peptide extracts, thereby revealing the cell destruction mechanism exerted.

## 2. Results and Discussion

### 2.1. Identification of Peptides from S. nigra Flowers

It is estimated that around 80% of the world population uses natural products for primary health care purposes [2]. Scientific research supports the biological activity of many natural phytochemicals; in fact, several naturally derived plant substances show an extensive spectrum of biological properties, including anti-oxidant, anti-bacterial, anti-viral, and anti-inflammatory activity, among others [16]. Thus, plants are a source of biotech products. 

**Figure 1:**
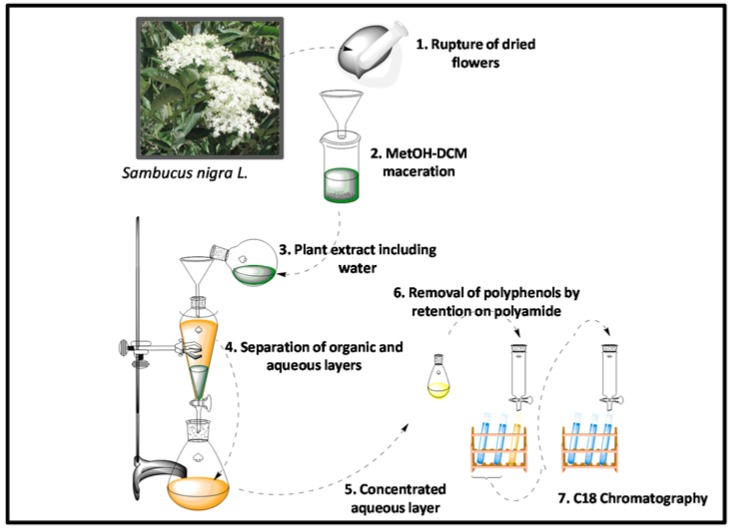
Schematic representation of the major steps for the peptide extraction procedure.

Plant AMPs are a component of the defense system against phyto-pathogens. However, these peptides also show antimicrobial activity against various human pathogens, and therefore emerge as promising antibiotic compounds with important biotechnological applications [7]. In the present study, we demonstrate the presence of AMPs in flowers of *S. nigra* L., which is a popular medicinal plant that is used in South America. The experimental procedure for peptide extraction is shown in Figure 1. The isolation and purification of peptides from plants can be complicated by their propensity to degrade when exposed to solvents [10]. Nevertheless, CRPs from plants show exceptional resistance to thermal/chemical denaturation [8]. 

Here, our strategy involved immersing the plant material in a DCM-MeOH mixture (1:1, *v*/*v*) and leaving it overnight at room temperature, a procedure that is widely used for peptide extraction [10]. However, after water addition, the aqueous layer contained large amounts of polyphenols (0.987 mg/mL). In fact, elder trees contain elevated amounts of polyphenolic compounds [17,18,19,20]. Thus, polyamide resin should be used for their removal because strong hydrogen bonding occurs between polyphenolics and polyamide, but peptides are not retained on this column support [21].

**Figure 2:**
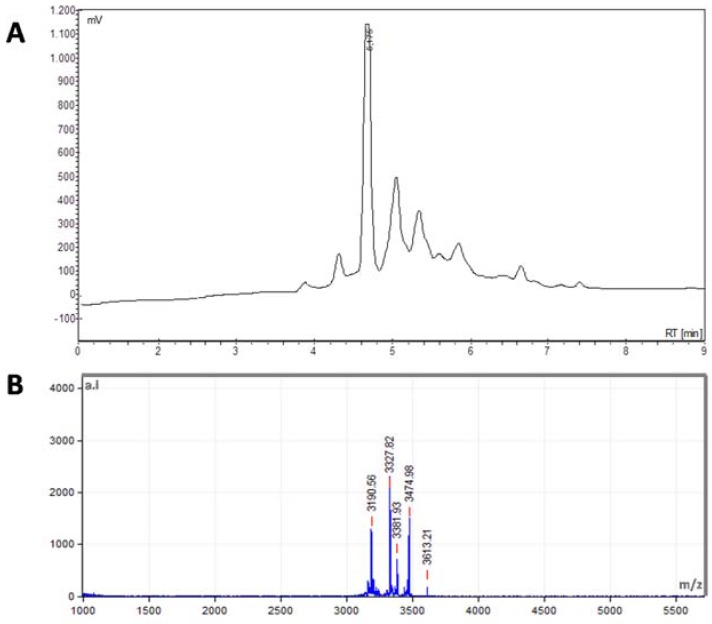
Characterization of peptide extract from flowers of *S. nigra*. (**A**) HPLC spectra of 40% acetonitrile (ACN) fraction of flowers peptide extract; (**B**) Matrix-Assisted Laser Desorption Ionization-Time of Flight Mass Spectrometer (MALDI-TOF) MS spectra of the 40% ACN fraction of flowers peptide extract.

Analytical reversed-phase (RP)-HPLC chromatogram of a peptide extract from *S. nigra* flowers after solvent extraction and in-batch C18 purification shows multiple peptide peaks (Figure 2A), and molecular masses between 3.1 and 3.6 kDa were determined for them by Matrix-Assisted Laser Desorption Ionization-Time of Flight Mass Spectrometer (MALDI-TOF) spectrometry (Figure 2B).

Cyclotides are head-to-tail CRPs with typical masses of 2–4 kDa [10,22,23,24], thereby suggesting the presence of cyclotide-like peptides in the *S. nigra* flowers. Nevertheless, the structure that is adopted by cyclotides prohibits direct fragmentation analysis. Thus, a partial primary structure of peptides from *S. nigra* flowers was determined by means of enzymatic fragmentation of reduced and alkylated peptides because these chemical modifications are necessary to yield precursor ions that are amenable to MS/MS sequencing [25,26,27]. The alkylated peptides were then cleaved with trypsin or endoproteinase Glu-C, and the resulting peptide fragments were identified by ESI-MS-MS mass spectrometry using Mascot and PEAKS servers. The partial characterization of AMPs identified from *S. nigra* flowers is presented in Table 1. 

From the analysis of the MS/MS data the identification of a peptide as chassatide C10 is based on a match with a small part of the sequence described for it in the literature for this (5/29 = 17% sequence coverage), but this identification was complemented with MALDI-MS data where the signal *m*/*z* 3212.107 was detected showing an error of −55.7 ppm with respect to the expected m/z for this cyclotide. Similarly, from the MS/MS data, a peptide was identified as glopa E based on a coincidence with part of the sequence described for it (6/30 = 20% sequence coverage); in addition, the MALDI-MS data suggest the presence of a peptide with a *m*/*z* of 3228.618 close to the expected value for glopa E of *m*/*z* 3227.398. In both cases it would be necessary to detect the missing tryptic peptide to confirm the complete sequence.

The identification of tryptic peptides as the cyclotides caripe 4 and vaby C was based on the sequence matching of 59 and 45% respectively, as determined from the analysis of the MS/MS data. Finally, the identification as phyb A was based on the coincidence of 83% of the sequence. In summary, MALDI-MS data show that the detected peptides are within the *m*/*z* range described for cyclotides while the MS/MS data showed partial coincidence with sequences of known cyclotides. However, more studies are necessary for the characterization of the primary and secondary structures of these molecules identified in the flowers of *S. nigra*.

### 2.2. Antimicrobial Activity of S. nigra Flowers Peptides

Antimicrobial peptides from plants, including Cyclotides, were initially studied because their main function is the control of opportunistic pathogens [28]. Given their broad antimicrobial spectrum, these molecules emerge as interesting targets to be exploited for the improvement of animal health. Here, we focused on the study of the antimicrobial activity of peptide extract from *S. nigra* flowers against various Gram-negative bacterial pathogens that affect fish aquaculture.

**Figure 3:**
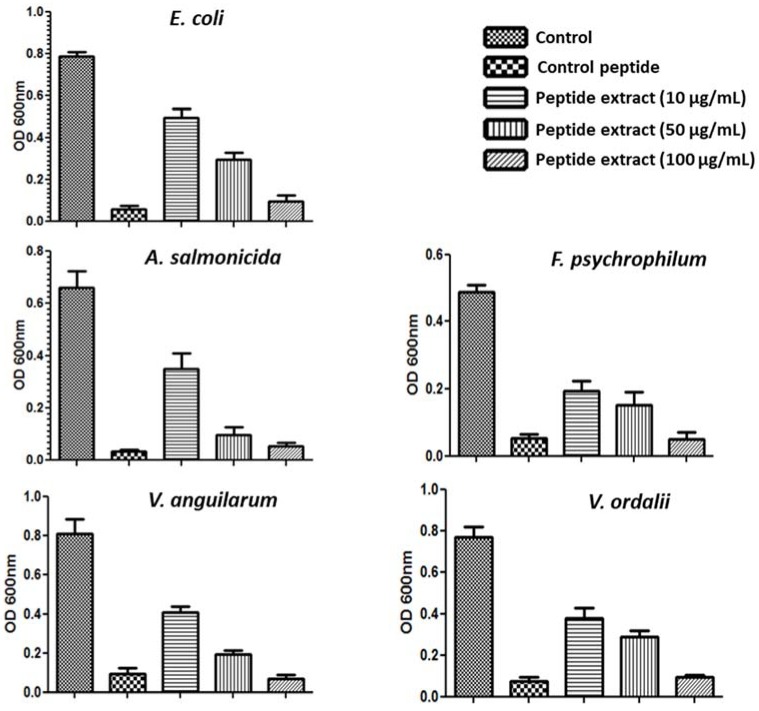
Antimicrobial activity of the *S. nigra* peptide extract. Microplate antimicrobial assay of peptide extract against *E. coli*, *A. salmonicida* sp. *salmonicida*, *F. psychrophilum*, *V. anguillarum* and *V. ordalii*. Antibacterial activity was evaluated with 10 μg/mL, 50 μg/mL, or 100 μg/mL of the flowers peptide extract (*n* = 6) in two independent experiment. Phospholipase-A2-derived synthetic peptide at 55.7 μg/mL was used as a positive control. Negative controls were performed under the same conditions without the addition of peptide.

Antimicrobial resistance in traditional fish farming has been widely studied [29,30,31,32]. Fish farms are an environmental reservoir of antibiotic resistance genes, because excess food containing antibiotic is deposited in the seabed. In this regard, residues of antimicrobials have been found in the sediments of marine fish farms [33]. Furthermore, 80 gram-negative strains that were isolated from water samples of Chilean salmonid farms have been reported [34]. For this reason, some plant extracts have been evaluated against a number of fish bacterial pathogens [35,36,37]. Here, the activity of the peptide extract from *S. nigra* flowers was evaluated against *A. salmonicida*, *F. psychrophilum*, *V. anguillarum,* and *V. ordalii*, all of them Gram-negative bacterial pathogens that were found in salmon aquaculture in Chile. Microplate assays showed the capacity of the extract to reduce the growth of all pathogens (Figure 3). The strongest antimicrobial effect was observed at a concentration of 100 μg/mL of the peptide extract.

### 2.3. Bacterial Membrane Damage Induced by S. nigra Flowers Peptides

**Figure 4:**
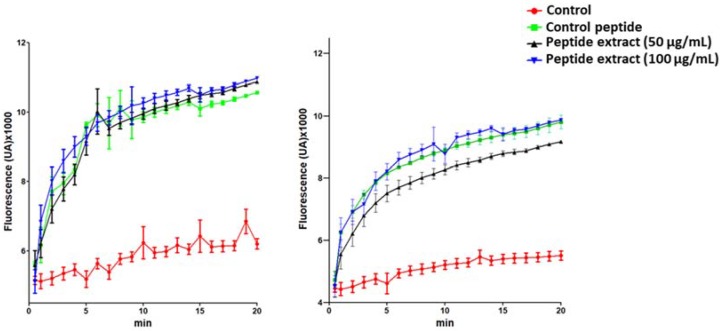
Membrane permeabilization influx of SYTOX Green in *E. coli* and *A. salmonicida* cells. The bacteria were exposed with 50 μg/mL or 100 μg/mL of the flowers peptide extract for 20 min in the presense of 5 μM SYTOX Green. Phospholipase-A2-derived synthetic peptide at 55.7 μg/mL was used as a positive control. Negative controls were performed under the same conditions without the addition of peptide. The increase in fluorescence was recorded at 30 s intervals with SYBR green filter.

One of the few conserved characteristics of AMPs is their cationic and hydrophobic composition [38]. This makes them well suited for interacting with anionic surfaces of microbial membranes, which typically present a high content of lipids, such as phosphatidylglycerol, cardiolipin, lypopolysaccharides, and teichoic acids [39,40,41]. Several models for the interaction of AMPs with the membranes, such as “barrel stave”, “toroidal pore”, or “carpet model” have been postulated [42]. In the carpet model, the peptides form a layer of “carpet” that induces membrane weakness, which ultimately ends with membrane collapse by a detergent-like action. According to this mechanism, peptides affect the local curvature of the bilayer in a cooperative manner, such that a toroid of high curvature is formed [43,38]. The effect of the peptide extract from *S. nigra* on the bacterial membrane integrity of *E. coli* and *A. salmonicida* was analyzed. This is usually studied by using a fluorescent nucleic acid stain, such as SYTOX Green, which is impervious to living cells [44]. Phospholipase-A2-derived synthetic peptide was used as a positive control, because its action on bacterial membranes has been previously described [45]. As expected, a strong increase in fluorescence occurred after treatment with the control peptide for 2 min (Figure 4). Interestingly, treatment with 50 μg/mL or 100 μg/mL of peptide extract showed similar results, with early increasing fluorescence (Figure 4). 

**Figure 5:**
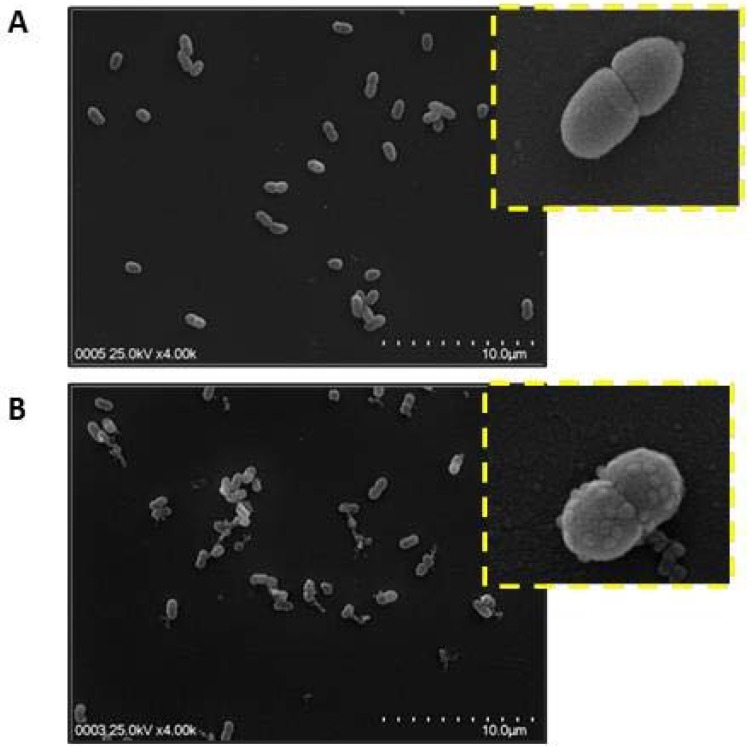
Bacterial membrane damage induced by *S. nigra* peptide extract. Scanning electron microscopy (SEM) micrographs of *A. salmonicida* sp. *salmonicida* without peptide (**A**) and with the presence of 50 μg/mL of the flowers peptide extract (**B**). Segmented yellow quadrate shows a zoom of representative bacteria.

In addition, a direct visualization of bacterial membrane damage following treatment with the peptide extract was obtained by scanning electron microscopy (SEM) (Figure 5). The SEM images of *A. salmonicida* (at mid-logarithmic growth phase) that were treated with the peptide extract showed membrane damage with multiple blisters. Moreover, intracellular contents were released in the bacteria, accompanied by membrane blebbing, suggesting membrane disruption as the mechanism of action of these peptides (Figure 5B). Thus, the SEM images suggest that peptides from *S. nigra* flowers use a carpet mechanism to kill bacteria, because the presence of membrane blebbing is associated with this model [46,47,48]. Nevertheless, future studies are necessary for identifying interactions of AMP from *S. nigra* flowers with the bacterial membrane components to understanding the antimicrobial mechanisms of these AMPs [49].

## 3. Materials and Methods 

### 3.1**.** Peptide Extraction

Flowers from *S. nigra* (Loncoche, IX region, Chile) were dried and disintegrated in a blender. They were then weighed (80 g) and homogenized in a mixture of dicloromethane (DCM)/methanol (MeOH) (1:1) (2 mL per g) [11]. The extract was filtered and transferred to a separating funnel. After that, UPW-Ultra Pure Water was added (2 mL per 10 mL of extract), and the solution was mixed. The organic (lower) layer was discarded and the aqueous layer was collected and placed on a rotary evaporator to remove MeOH. The extract was freeze-dried and weighed. The dried extract was then reconstituted with water and was passed through a DPA-6S polyamide SPE cartridge (Sigma-Aldrich, St. Louis, MO, USA) to remove polyphenols and other compounds. The aqueous elution was applied onto a Sep-pak C18 Vac cartridge (Waters Associates, Milford, MA, USA) and equilibrated in acidified water (0.05% trifluoroacetic (TFA) acid in UPW-Ultra Pure Water). After washing with acidified water, the peptides were eluted at a flow-rate of 1 mL/min with 5%, 10%, 20%, 30%, 40%, 60%, and 80% acetonitrile (ACN). The appropriate fractions were collected, and the ACN was evaporated on a speedvac centrifugal device. The fractions were then analyzed by reversed-phase (RP)-HPLC (Waters Associates, Milford, MA, USA) on a Water Corp XBridge™ BEH C18 column (100 × 4.6 mm, 3.5 μm) Waters Associates, Milford, MA, USA) using a 0–70% ACN gradient, water containing 0.05% TFA as solvent A, and ACN containing 0.05% TFA as solvent B, at a flow rate of 1 mL/min for 8 min. 

### 3.2**.** Mass Spectrometry Analysis for Peptide Identification

The acquisition of mass spectra of each ACN fraction was performed in a Matrix-Assisted Laser Desorption Ionization-Time of Flight Mass Spectrometer (MALDI-TOF) Microflex (Bruker Daltonics Inc., Billerica, MA, USA). The 40% ACN fraction was then prepared for ESI MS/MS sequencing as described earlier [50,51]. Briefly, the extract was reduced (dithiothreitol), alkylated with iodoacetamide, and enzymatically digested using trypsin or endo-GluC (Sigma-Aldrich and Promega Corp., Madison, WI, USA, respectively) [51]. The proteolyzed samples were examined in an LC-MS-MS system consisting of an Agilent 1100 HPLC (Agilent Technologies Inc., Santa Clara, CA, USA) coupled to a ESI-TRAP Esquire 4000 ion-trap type mass spectrometer (Bruker Daltonik GmbH, Bremen, Germany). For the analysis of the chromatograms and LC-ESI-MS-MS, DataAnalysis version 3.2 software (Bruker Daltonik GmbH, Bremen, Germany) was used. Mascot Server version 2.0 (Matrix Science, London, UK), PEAKS Studio version 8.0 (Bioinformatics Solutions Inc., Waterloo, ON, Canada) and CyBase [52,53] were used for the peptide identification.

### 3.3. Antibacterial Assay 

Antibacterial activity was determined using the microplate assay, as previously described [54,55,56,57]. Three concentrations of the peptide extract were tested to evaluate antibacterial activity. 10 μg/mL, 50 μg/mL and 100 μg/mL of peptide extract (40% ACN fraction) was mixed with 100 μL of an exponential phase bacterial culture of *Escherichia coli*, *Vibrio anguillarum*, *Vibrio ordalii*, *Flavobacterium psychrophilum*, and *Aeromonas salmonicida*. In addition, phospholipase-A2-derived synthetic peptide variant was used as a positive control [47]. The test was performed at a starting OD of 0.001 at 620 nm in tryptic soy broth (TSB) for *E. coli* and *A. salmonicida*, TSB containing 1.5% NaCl for *V. anguillarum* or and Anacker and Ordal’s (AOAE) liquid medium for *F. psychrophilum*. After 16 h of incubation (37 °C for E. coli; 24 °C for *A. salmonicida subs salmonicida*, and *Flavobacterium psychrophilum*; 25 °C for *V. anguillarum* and *Vibrio ordalii*), absorbance values were measured [58,59].

### 3.4. SYTOX Green Bacteria Permeabilization Assay

SYTOX Green uptake assay was performed, according to a previously described procedure [58]. Cultures of exponentially-grown *E. coli* and *A. salmonicida* were diluted in 10 mM sodium phosphate buffer pH 7.2 to a cell density of 1 × 10^6^ CFU/mL. Then, aliquots of 90 μL of these cell cultures were deposited in optics real time PCR tubes and 5 μL of the solution of peptide extract (50 and 100 μg/mL) and 5 μL of 100 μM SYTOX Green were added to the wells, and then the tubes were placed in a thermocycler (Agilent Mx3000p qPCR System, Agilent Technologies, Santa Clara, CA, USA). The thermocycler program was performed using the SYBR green filter selected, 40 cycles of 30 s at 37 °C (*E. coli*) or 24 °C (*A. salmonicida*), with reading at the end of each cycle. Control experiments were performed under the same conditions without the addition of peptide. Independent experiments were repeated a minimum of three times.

### 3.5**.** Scanning Electron Microscopy (SEM)

*A. salmonicida* was cultured in TSB medium to the mid-log phase and was harvested by centrifugation at 1000× *g* for 5 min. Cell pellets were washed twice with 10 mM PBS and resuspended to an OD_600_ nm of 0.3. The cell suspension was incubated at 37 °C for 30 min with 50 μg/mL of peptide extract or phospholipase-A2-derived synthetic peptide at 20 μM. After incubation, the cells were washed three times with PBS. Bacterial pellets were then fixed overnight in 500 μL of 2.5% (*v*/*v*) glutaraldehyde in PBS at 4 °C. Subsequently, the bacterial samples were dehydrated with graded ethanol series and then dried. A small amount of platinum was sputtered on the samples to avoid charging in the microscope. Cells were examined under a scanning electron microscope (Hitachi SU 3500, Tokyo, Japan).

### 3.6. Polyphenol Quantification

The content of total phenols of *S. nigra* extracts was measured by Folin-Ciocalteu spectrophotometry assay, according to the technique of Singleton and Rossi (1965) [60]. Methanol solutions of gallic acid at concentrations ranging between 100 and 1000 μg/mL were used as calibration curve. 5 μL of each gallic acid solutions and *S. nigra* extracts were used for the quantification of phenols. 75 μL of distilled water and 20 μL of 1 N Folin-Coicalteu reagent were added to each. After 3 min, 30 μL of Na_2_CO_3_ solution (10% *w*/*v*) was added and then 120 μL with distilled water were added. After 2 h of dark incubation, the absorbance at 760 nm was measured in a microplate reader. The content of total phenols was expressed as mg/mL.

## 4. Conclusions

AMPs are emerging as therapeutic alternatives to conventional antibiotics. Medicinal plant extracts are a plentiful source of novel agents and could be exploited in different fields, such as in animal farming infections control. The presence of cysteine-rich peptides in flowers of *S. nigra* has been determined that exhibit activity against different fish pathogens of interest in Chilean aquaculture. It is postulated that their antimicrobial activity is related to the disruption of the bacterial cell membrane. However, further studies are needed to characterize the structure and activity of each peptide present in the flowers of *S. nigra*.

## Figures and Tables

**Table 1:** Partial characterization of antimicrobial peptides of *S. nigra* flowers by LC−MS/MS Peptide sequencing.

Detected Sequence ^a^	Reported Cysteine-Rich Peptides (CRP)	Family CRP
GEYCGESCYLIPCFTPGCYCVSRQCVNKN ^b^	chassatide_C10 (*Chassalia_chartacea*)	Cyclotide
GIPCAESCVWIPCTVTKMLGCSCKDKVCYN ^c^	Glopa E (*Gloeospermum pauciflorum Hekking*)	Cyclotide
LICSSTCLRIPCSPRCTVRHHICYLN ^b^	Caripe 4 (*Carapichea Ipecacuanha*)	Cyclotide
GLPVCGETCAGGRCNTPGCSCSWPVCTRN ^b^	Vaby C (*viola abyssinica*)	Cyclotide
GIGCGESCVWIPCVSAAIGCSCSNKICYRN ^b^	Phyb_A (*Petunia hybrida*)	Cyclotide

^a^ Identified peptidic fragments are showed in red; ^b^ Identified in trypsin digest. ^c^ Identified in endoproteinase GluC digest.

## Supplementary Materials

Supplementary File 1
