# Supplementary material for: Identification of Peptides in Flowers of Sambucus nigra with Antimicrobial Activity against Aquaculture Pathogens
Source: Molecules. 2018 Apr 27;23(5):1033. doi: 10.3390/molecules23051033 (PMC6100021; doi:10.3390/molecules23051033)
Supplement: Supplementary File 1 [file molecules-23-01033-s001.pdf]

molecules

MDPI

Article

Identification of Peptides in Flowers of *Sambucus nigra* with Antimicrobial Activity against Aquaculture Pathogens

Claudio Andrés Álvarez [1,2,3], Andrés Barriga [4], Fernando Albericio [5,6], María Soledad Romero [2] and Fanny Guzmán [7,*]

[1] Laboratorio de Fisiología y Genética Marina (FIGEMA), Centro de Estudios Avanzados en Zonas Áridas (CEAZA), Coquimbo 1781421, Chile; claudio.alvarez@ceaza.cl
[2] Facultad de Ciencias del Mar, Universidad Católica del Norte, Coquimbo 1781421, Chile
[3] Centro AquaPacífico, Coquimbo 1781421, Chile
[4] Unidad de Espectrometría de Masas, Facultad de Ciencias Químicas y Farmacéuticas, Universidad de Chile, Santiago 8380494, Chile; anbarr@ciq.uchile.cl
[5] Department of Organic Chemistry and CIBER-BBN, Networking Centre on Bioengineering, Biomaterials and Nanomedicine, University of Barcelona, Barcelona 08007, Spain; albericio@ukzn.ac.za
[6] School of Chemistry, University of KwaZulu-Natal, Durban 4001, South Africa
[7] Núcleo de Biotecnología de Curauma, Pontificia Universidad Católica de Valparaíso, Valparaíso 2373223, Chile
* Correspondence: fanny.guzman@pucv.cl; Tel.: +56-32-2274827

Received: 27 March 2018; Accepted: 26 April 2018; Published: date

Abstract: The elder (*Sambucus* spp.) tree has a number of uses in traditional medicine. Previous studies have demonstrated the antimicrobial properties of elderberry liquid extract against human pathogenic bacteria and also influenza viruses. These properties have been mainly attributed to phenolic compounds. However, other plant defense molecules, such as antimicrobial peptides (AMPs), may be present. Here, we studied peptide extracts from flowers of *Sambucus nigra* L. The mass spectrometry analyses determined peptides of 3 to 3.6 kDa, among them, cysteine-rich peptides were identified with antimicrobial activity against various Gram-negative bacteria, including recurrent pathogens of Chilean aquaculture. In addition, membrane blebbing on the bacterial surface after exposure to the cyclotide was visualized by SEM microscopy and SYTOX Green permeabilization assay showed the ability to disrupt the bacterial membrane. We postulate that these peptides exert their action by destroying the bacterial membrane.

Keywords: *S. nigra*; peptides; antimicrobial activity; fish pathogens; membrane damage

1. Introduction

Herbal medicine accounts for a considerable share of the pharmaceutical market. In this regard, the global market value of medicinal plant products exceeds US$7 billion a year [1,2]. One medicinal plant is the elder tree, whose berries are widely used in herbal medicine. Previous studies have demonstrated that these berries contain various compounds with high biological activity, such as flavonoids, proanthocyanidins, anthocyanins, and phenolic acids [3,4]. These compounds confer protection against oxidative stress that is caused by reactive oxygen species, which are known to be involved in disorders, like cancer and hypertension [5]. Moreover, elderberry liquid extract shows activity against human pathogenic bacteria and also influenza viruses [6].

Comment [M1]: The names are different from redmine, please check.
Comment [M2]: Please carefully check the accuracy of names and affiliations. Changes will not be possible after proofreading.
Formatted: Font:Palatino Linotype, 10 pt, Bold, Superscript
Comment [M3]: The order is different from redmine, please check.
Deleted: ,7
Deleted: 8
Deleted: 4,5
Formatted: Font:Palatino Linotype, 10 pt, Bold, Superscript
Comment [M5]: Please add city, post code and country.
Comment [c6]: The post code were included
Formatted: Font:Palatino Linotype, 9 pt, Not Highlight
Formatted: Font:Palatino Linotype, 9 pt
Deleted: ;
Formatted: Font:Palatino Linotype, 9 pt, Not Highlight
Comment [M7]: Please provide more detailed university information, like department.
Formatted: Font:Palatino Linotype, 9 pt
Deleted: Coquimbo,
Formatted: Font:Palatino Linotype, 9 pt, Not Highlight
Formatted: Font:Palatino Linotype, 9 pt
Deleted: Coquimbo
Moved down [1]: 5 Núcleo de Biotecnología de Curauma, Pontificia Universidad Católica de Valparaíso, Valparaíso, Chile
Deleted: 6
Formatted: Font:Palatino Linotype, 9 pt, Not Highlight
Formatted: Font:Palatino Linotype, 9 pt, English (US)
Deleted: 7
Formatted: Font:Palatino Linotype, 9 pt, Not Highlight
Formatted: Font:Palatino Linotype, 9 pt, English (US)
Deleted: 8 Departamento de Acuicultura, Facultad ... [1]
Formatted: Not Highlight
Moved (insertion) [1]
Deleted: 5
Formatted: Font:Palatino Linotype, 9 pt, Not Highlight
Formatted: Font:Palatino Linotype, 9 pt
Formatted: Font:Palatino Linotype, 9 pt, Not Highlight
Comment [M9]: Change hyphen to ; please confirm. ... [2]
Comment [c10]: It is right.

In addition to these compounds, antimicrobial peptides (AMPs) perform a key role in plant defense against pathogens [7]. Plant AMPs are generally rich in cysteine residues that form multiple disulfides. Cysteine-rich peptides (CRP) families include thionins, defensins, hevein-like peptides, cyclotides, lipid transfer proteins, and the α-hairpinin and snakins family [8–10]. In this regard, studies have addressed the use of plant AMPs as substitutes of chemical preservatives and insecticides in agriculture applications [11,12]. In addition, the features of these antimicrobial molecules make them suitable for use in animal farming and public health, thereby decreasing the use of antibiotics.

Plant AMPs have been isolated and characterized in roots, leaves, and seeds, although they are also present in flower tissues [13]. AMPs from flowers have been characterized on the basis of cationic charge, thereby pointing to lipid bilayer permeabilization as a possible action mechanism [13]. However, given that flowers have received less attention than other tissues, they may hold a number of as yet undiscovered AMPs with biotechnological applications.

Herbal medicine is a long-standing tradition in the Mapuche communities of southern Argentina and Chile [14]. Among the plants that are used for this purpose is *Sambucus nigra* L. (common name "Sauco" in Spanish), which bears berries that contain anti-oxidant phytonutrients that are similar to those found in other elderberries [15]. However, the antimicrobial properties of this medicinal plant have not been established to date. Here, we developed an experimental procedure to obtain a peptide extract from *S. nigra* flowers. Cysteine-rich peptides (CRPs) were identified by mass spectrometry, and the peptide extracts were analyzed for antimicrobial properties, including activity against pathogen bacteria that affect Chilean aquaculture. In addition, scanning electron microscopy (SEM) assays were performed in order to directly observe the response of bacterial cell morphology and membrane integrity to the treatment with peptide extracts, thereby revealing the cell destruction mechanism exerted.

2. Results and Discussion

2.1. Identification of Peptides from S. nigra Flowers

It is estimated that around 80% of the world population uses natural products for primary health care purposes [2]. Scientific research supports the biological activity of many natural phytochemicals; in fact, several naturally derived plant substances show an extensive spectrum of biological properties, including anti-oxidant, anti-bacterial, anti-viral, and anti-inflammatory activity, among others [16]. Thus, plants are a source of biotech products.

Plant AMPs are a component of the defense system against phyto-pathogens. However, these peptides also show antimicrobial activity against various human pathogens, and therefore emerge as promising antibiotic compounds with important biotechnological applications [7]. In the present study, we demonstrate the presence of AMPs in flowers of *S. nigra* L., which is a popular medicinal plant that is used in South America. The experimental procedure for peptide extraction is shown in Figure 1. The isolation and purification of peptides from plants can be complicated by their propensity to degrade when exposed to solvents [10]. Nevertheless, CRPs from plants show exceptional resistance to thermal/chemical denaturation [8].

Here, our strategy involved immersing the plant material in a DCM-MeOH mixture (1:1, *v*/*v*) and leaving it overnight at room temperature, a procedure that is widely used for peptide extraction [10]. However, after water addition, the aqueous layer contained large amounts of polyphenols (0.987 mg/mL). In fact, elder trees contain elevated amounts of polyphenolic compounds [17–20]. Thus, polyamide resin should be used for their removal because strong hydrogen bonding occurs between polyphenolics and polyamide, but peptides are not retained on this column support [21].

Deleted: 17

Deleted: 17

Deleted: 8

Deleted: 1

Deleted: 2

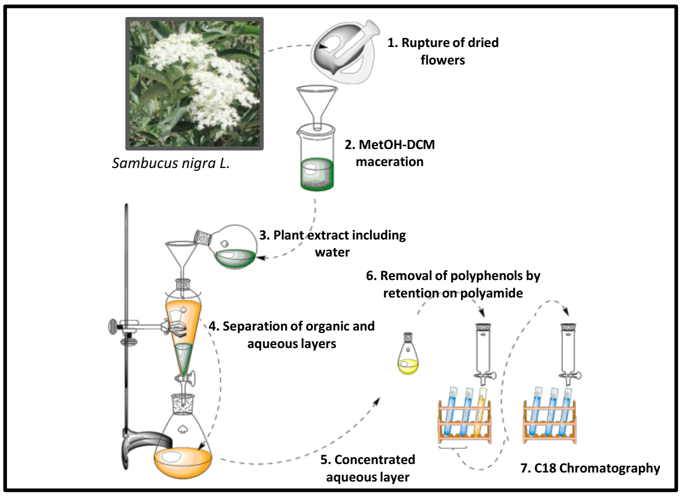

Figure 1. Schematic representation of the major steps for the peptide extraction procedure.

Analytical reversed-phase (RP)-HPLC chromatogram of a peptide extract from *S. nigra* flowers after solvent extraction and in-batch C18 purification shows multiple peptide peaks (Figure 2A), and molecular masses between 3.1 and 3.6 kDa were determined for them by Matrix-Assisted Laser Desorption Ionization-Time of Flight Mass Spectrometer (MALDI-TOF) spectrometry (Figure 2B).

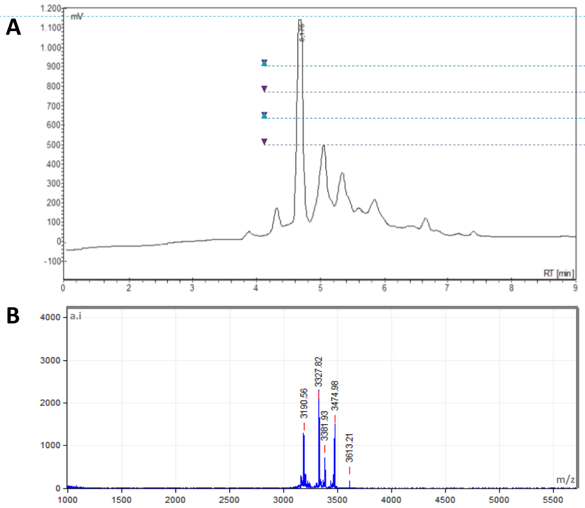

Figure 2. Characterization of peptide extract from flowers of *S. nigra*. (**A**) HPLC spectra of 40% acetonitrile (ACN) fraction of flowers peptide extract; (**B**) Matrix-Assisted Laser Desorption Ionization-Time of Flight Mass Spectrometer (MALDI-TOF) MS spectra of the 40% ACN fraction of flowers peptide extract.

Comment [M11]: There is not Figure 2C.

Comment [c12]: This mistake was corrected.

Deleted: C

Formatted: Font:Palatino Linotype, 10 pt

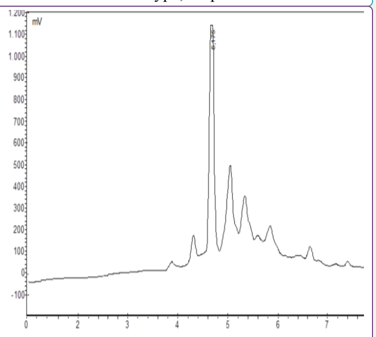

Deleted:

Formatted: Font:Palatino Linotype, 10 pt

Deleted: (A)

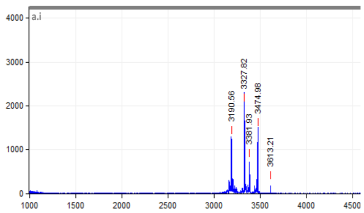

Deleted:

Formatted: Font:Palatino Linotype, 10 pt

Deleted: (B)

Comment [M13]: Please replace (A) with a sharper image.

Comment [c14]: The image was modified.

Cyclotides are head-to-tail CRPs with typical masses of 2–4 kDa [10,22–24], thereby suggesting the presence of cyclotide-like peptides in the *S. nigra* flowers. Nevertheless, the structure that is adopted by cyclotides prohibits direct fragmentation analysis. Thus, a partial primary structure of peptides from *S. nigra* flowers was determined by means of enzymatic fragmentation of reduced and alkylated peptides because these chemical modifications are necessary to yield precursor ions that are amenable to MS/MS sequencing [25–27]. The alkylated peptides were then cleaved with trypsin or endoproteinase Glu-C, and the resulting peptide fragments were identified by ESI-MS-MS mass spectrometry using Mascot and PEAKS servers. The partial characterization of AMPs identified from *S. nigra* flowers is presented in Table 1.

From the analysis of the MS/MS data the identification of a peptide as chassatide C10 is based on a match with a small part of the sequence described for it in the literature for this (5/29 = 17% sequence coverage), but this identification was complemented with MALDI-MS data where the signal *m*/*z* 3212.107 was detected showing an error of −55.7 ppm with respect to the expected m/z for this cyclotide. Similarly, from the MS/MS data, a peptide was identified as glopa E based on a coincidence with part of the sequence described for it (6/30 = 20% sequence coverage); in addition, the MALDI-MS data suggest the presence of a peptide with a *m*/*z* of 3228.618 close to the expected value for glopa E of *m*/*z* 3227.398. In both cases it would be necessary to detect the missing tryptic peptide to confirm the complete sequence.

The identification of tryptic peptides as the cyclotides caripe 4 and vaby C was based on the sequence matching of 59 and 45% respectively, as determined from the analysis of the MS/MS data. Finally, the identification as phyb A was based on the coincidence of 83% of the sequence. In summary, MALDI-MS data show that the detected peptides are within the *m*/*z* range described for cyclotides while the MS/MS data showed partial coincidence with sequences of known cyclotides. However, more studies are necessary for the characterization of the primary and secondary structures of these molecules identified in the flowers of *S. nigra*.

Table 1. Partial characterization of antimicrobial peptides of *S. nigra* flowers by LC−MS/MS Peptide sequencing.

Detected Sequence [a]	Reported Cysteine-Rich Peptides (CRP)	Family CRP
GEYCGESCYLIPCFTPGCYCVSRQCVNKN [b]	chassatide_C10 (*Chassalia chartacea*)	Cyclotide
GIPCAESCVWIPCTVTKMLGCSCKDKVCYN [c]	Glopa E (*Gloeospermum pauciflorum Hekking*)	Cyclotide
LICSSTCLRIPCSPRCTVRHHICYLN [b]	Caripe 4 (*Carapichea Ipecacuanha*)	Cyclotide
GLPVCGETCAGGRCNTPGCSCSWPVCTRN [b]	Vaby C (*viola abyssinica*)	Cyclotide
GIGCGESCVWIPCVSAAIGCSCSNKICYRN [b]	Phyb_A (*Petunia hybrida*)	Cyclotide

[a] Identified peptidic fragments are showed in red. [b] Identified in trypsin digest. [c] Identified in endoproteinase GluC digest.

2.2. Antimicrobial Activity of S. nigra Flowers Peptides

Antimicrobial peptides from plants, including Cyclotides, were initially studied because their main function is the control of opportunistic pathogens [28]. Given their broad antimicrobial spectrum, these molecules emerge as interesting targets to be exploited for the improvement of animal health. Here, we focused on the study of the antimicrobial activity of peptide extract from *S. nigra* flowers against various Gram-negative bacterial pathogens that affect fish aquaculture.

Antimicrobial resistance in traditional fish farming has been widely studied [29–32]. Fish farms are an environmental reservoir of antibiotic resistance genes, because excess food containing antibiotic is deposited in the seabed. In this regard, residues of antimicrobials have been found in the sediments of marine fish farms [33]. Furthermore, 80 Gram-negative strains that were isolated from water samples of Chilean salmonid farms have been reported [34]. For this reason, some plant extracts have been evaluated against a number of fish bacterial pathogens [35–37]. Here, the activity of the peptide extract from *S. nigra* flowers was evaluated against *A. salmonicida*, *F. psychrophilum*, *V. anguillarum*, and *V. ordalii*, all of them Gram-negative bacterial pathogens that were found in salmon aquaculture in Chile. Microplate assays showed the capacity of the extract to reduce the growth of

Deleted: 17
Deleted: 3
Deleted: 5
Deleted: 6
Deleted: 8
Comment [M15]: Newly changed number, please confirm.
Comment [c16]: It is right, thanks
Comment [M17]: Change to minus sign, please confirm if it is right.
Comment [c18]: It is right, thanks
Comment [c19]: The color is necessary because indicates the peptidic fragment identified.
Comment [M20]: Is the Color necessary?
Comment [M22]: Whether the italics need to be added?
Resp: The italics is needed.
Deleted: [
Deleted:]
Formatted: Font:Palatino Linotype, Italic
Formatted: Font:Italic
Deleted: [
Deleted:]
Deleted: [
Deleted:]
Deleted: [
Deleted:]
Deleted: [
Deleted:]
Deleted: 9
Deleted: 30
Deleted: 3
Deleted: 4
Comment [c23]: It is right.
Comment [TC24]: Please confirm that this is correct.
Deleted: 5
Deleted: 6
Deleted: 8

all pathogens (Figure 3). The strongest antimicrobial effect was observed at a concentration of 100 μg/mL of the peptide extract.

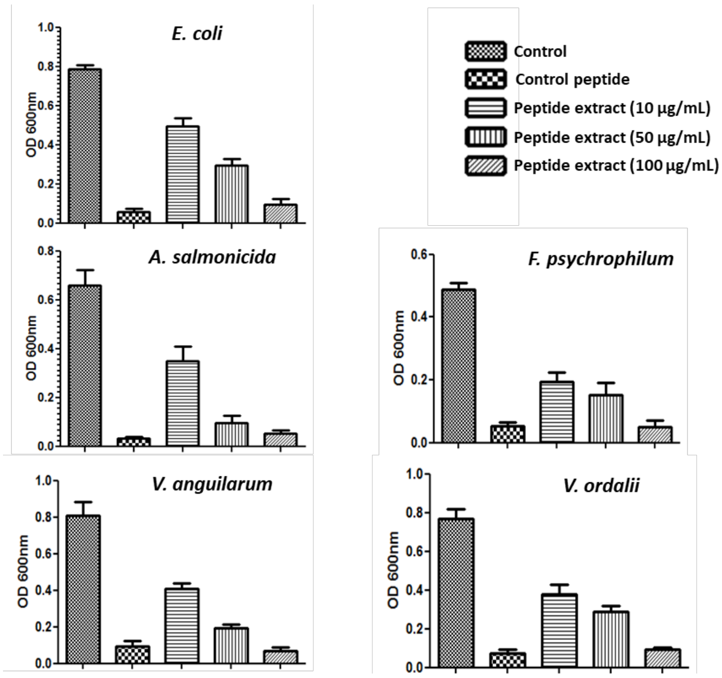

Figure 3. Antimicrobial activity of the *S. nigra* peptide extract. Microplate antimicrobial assay of peptide extract against *E. coli*, *A. salmonicida* sp. *salmonicida*, *F. psychrophilum*, *V. anguillarum* and *V. ordalii*. Antibacterial activity was evaluated with 10 μg/mL, 50 μg/mL, or 100 μg/mL of the flowers peptide extract (n = 6) in two independent experiment. Phospholipase-A2-derived synthetic peptide at 55.7 μg/mL was used as a positive control. Negative controls were performed under the same conditions without the addition of peptide.

2.3. Bacterial Membrane Damage Induced by S. nigra Flowers Peptides

One of the few conserved characteristics of AMPs is their cationic and hydrophobic composition [38]. This makes them well suited for interacting with anionic surfaces of microbial membranes, which typically present a high content of lipids, such as phosphatidylglycerol, cardiolipin, lypopolysaccharides, and teichoic acids [39–41]. Several models for the interaction of AMPs with the membranes, such as "barrel stave", "toroidal pore", or "carpet model" have been postulated [42]. In the carpet model, the peptides form a layer of "carpet" that induces membrane weakness, which ultimately ends with membrane collapse by a detergent-like action. According to this mechanism, peptides affect the local curvature of the bilayer in a cooperative manner, such that a toroid of high curvature is formed [43,38]. The effect of the peptide extract from *S. nigra* on the bacterial membrane integrity of *E. coli* and *A. salmonicida* was analyzed. This is usually studied by using a fluorescent nucleic acid stain, such as SYTOX Green, which is impervious to living cells [44]. Phospholipase-A2-derived synthetic peptide was used as a positive control, because its action on bacterial membranes has been previously described [45]. As expected, a strong increase in fluorescence occurred after treatment with the control peptide for 2 min (Figure 4). Interestingly, treatment with 50 μg/mL or 100 μg/mL of peptide extract showed similar results, with early increasing fluorescence (Figure 4).

Comment [M26]: Please confirm if it is correct. There is no A in the picture.

Deleted: (A

Deleted:)

Comment [M27]: Newly changed information, please confirm.

Comment [c28]: It is right.

Deleted: 9

Deleted: 40

Deleted: 2

Deleted: 3

Deleted: 4

Deleted: 45

Deleted: 6

Deleted: 7

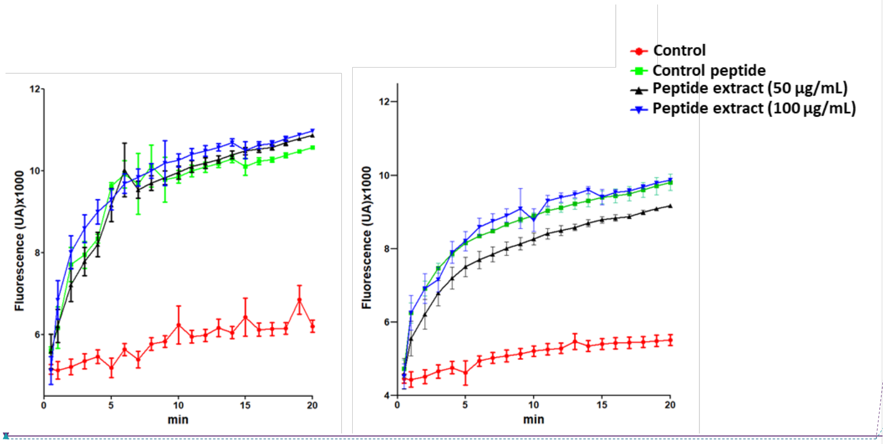

Figure 4. Membrane permeabilization influx of SYTOX Green in *E. coli* and *A. salmonicida* cells. The bacteria were exposed with 50 µg/mL or 100 µg/mL of the flowers peptide extract for 20 min in the presense of 5 µM SYTOX Green. Phospholipase-A2-derived synthetic peptide at 55.7 µg/mL was used as a positive control. Negative controls were performed under the same conditions without the addition of peptide. The increase in fluorescence was recorded at 30 s intervals with SYBR green filter.

In addition, a direct visualization of bacterial membrane damage following treatment with the peptide extract was obtained by scanning electron microscopy (SEM) (Figure 5). The SEM images of *A. salmonicida* (at mid-logarithmic growth phase) that were treated with the peptide extract showed membrane damage with multiple blisters. Moreover, intracellular contents were released in the bacteria, accompanied by membrane blebbing, suggesting membrane disruption as the mechanism of action of these peptides (Figure 5B). Thus, the SEM images suggest that peptides from *S. nigra* flowers use a carpet mechanism to kill bacteria, because the presence of membrane blebbing is associated with this model [46–48]. Nevertheless, future studies are necessary for identifying interactions of AMP from *S. nigra* flowers with the bacterial membrane components to understanding the antimicrobial mechanisms of these AMPs [49].

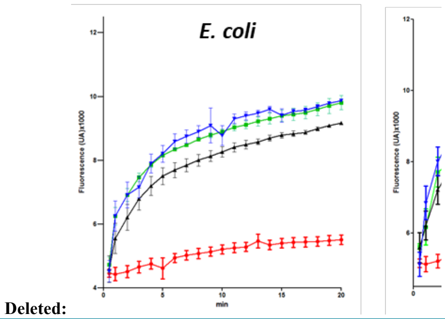

Deleted:

Formatted: Font:Palatino Linotype, 10 pt

Comment [M29]: Please replace with a sharper image. The word and number in x, y axis are too small. Please change.

Comment [c30]: The image was modified.

Deleted: 8

Deleted: 50

Deleted: 51

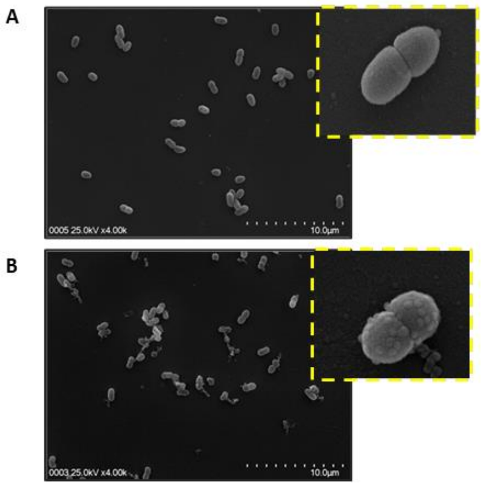

Figure 5. Bacterial membrane damage induced by *S. nigra* peptide extract. Scanning electron microscopy (SEM) micrographs of *A. salmonicida* sp. *salmonicida* without peptide (**A**) and with the presence of 50 µg/mL of the flowers peptide extract (**B**). Segmented yellow quadrate shows a zoom of representative bacteria.

3. Materials and Methods

3.1. Peptide Extraction

Flowers from *S. nigra* (Loncoche, IX region, Chile) were dried and disintegrated in a blender. They were then weighed (80 g) and homogenized in a mixture of diclorometháne (DCM)/methanol (MeOH) (1:1) (2 mL per g) [11]. The extract was filtered and transferred to a separating funnel. After that, UPW-Ultra Pure Water was added (2 mL per 10 mL of extract), and the solution was mixed. The organic (lower) layer was discarded and the aqueous layer was collected and placed on a rotary evaporator to remove MeOH. The extract was freeze-dried and weighed. The dried extract was then reconstituted with water and was passed through a DPA-6S polyamide SPE cartridge (Sigma-Aldrich, St. Louis, MO, USA) to remove polyphenols and other compounds. The aqueous elution was applied onto a Sep-pak C18 Vac cartridge (Waters Associates, Milford, MA 01757 USA) and equilibrated in acidified water (0.05% trifluoroacetic (TFA) acid in UPW-Ultra Pure Water). After washing with acidified water, the peptides were eluted at a flow-rate of 1 ml/min with 5%, 10%, 20%, 30%, 40%, 60%, and 80% acetonitrile (ACN). The appropriate fractions were collected, and the ACN was evaporated on a speedvac centrifugal device. The fractions were then analyzed by reversed-phase (RP)-HPLC (Waters Associates, Milford, MA 01757 USA) on a Water Corp XBridge™ BEH C18 column (100 × 4.6 mm, 3.5 µm) Waters Associates, Milford, MA 01757 USA) using a 0–70% ACN gradient, water containing 0.05% TFA as solvent A, and ACN containing 0.05% TFA as solvent B, at a flow rate of 1 mL/min for 8 min.

Comment [M31]: Newly added information, please confirm.
Comment [c32]: It is correct.
Deleted: company, city, country
Deleted: company, city, country
Deleted: (company, city, country

3.2. Mass Spectrometry Analysis for Peptide Identification

The acquisition of mass spectra of each ACN fraction was performed in a Matrix-Assisted Laser Desorption Ionization-Time of Flight Mass Spectrometer (MALDI-TOF) Microflex (Bruker Daltonics Inc., Billerica, MA, USA). The 40% ACN fraction was then prepared for ESI MS/MS sequencing as described earlier [50,51]. Briefly, the extract was reduced (dithiothreitol), alkylated with iodoacetamide, and enzymatically digested using trypsin or endo-GluC (Sigma-Aldrich and Promega Corp., Madison, WI, USA, respectively) [51]. The proteolyzed samples were examined in

Comment [M34]: Newly added information, please confirm.
Comment [c35]: It is right.
Deleted: 2
Deleted: 3
Deleted: (Colgrave et al., 2010)

an LC-MS-MS system consisting of an Agilent 1100 HPLC (Agilent Technologies Inc., Santa Clara, CA, USA) coupled to a ESI-TRAP Esquire 4000 ion-trap type mass spectrometer (Bruker Daltonik GmbH, Bremen, Germany). For the analysis of the chromatograms and LC-ESI-MS-MS, DataAnalysis version 3.2 software (Bruker Daltonik GmbH, Bremen, Germany) was used. Mascot Server version 2.0 (Matrix Science, London, UK), PEAKS Studio version 8.0 (Bioinformatics Solutions Inc., Waterloo, ON, Canada) and CyBase [52,53] were used for the peptide identification.

Deleted: [ERA cyclotide database tool
Deleted: 4
Deleted: 5

3.3. Antibacterial Assay

Antibacterial activity was determined using the microplate assay, as previously described [54–57]. Three concentrations of the peptide extract were tested to evaluate antibacterial activity. 10 μg/mL, 50 μg/mL and 100 μg/mL of peptide extract (40% ACN fraction) was mixed with 100 μL of an exponential phase bacterial culture of *Escherichia coli*, *Vibrio anguillarum*, *Vibrio ordalii*, *Flavobacterium psychrophilum*, and *Aeromonas salmonicida*. In addition, phospholipase-A2-derived synthetic peptide variant was used as a positive control [47]. The test was performed at a starting OD of 0.001 at 620 nm in tryptic soy broth (TSB) for *E. coli* and *A. salmonicida*, TSB containing 1.5% NaCl for *V. anguillarum* or and Anacker and Ordal's (AOAE) liquid medium for *F. psychrophilum*. After 16 h of incubation (37 °C for E. coli; 24 °C for *A. salmonicida subs salmonicida*, and *Flavobacterium psychrophilum*; 25 °C for *V. anguillarum* and *Vibrio ordalii*), absorbance values were measured [58,59].

Deleted: 6
Deleted: 9
Deleted: 60
Deleted: 61

3.4. SYTOX Green Bacteria Permeabilization Assay

SYTOX Green uptake assay was performed, according to a previously described procedure [58]. Cultures of exponentially-grown *E. coli* and *A. salmonicida* were diluted in 10 mM sodium phosphate buffer pH 7.2 to a cell density of 1 × 10^6 CFU/mL. Then, aliquots of 90 μL of these cell cultures were deposited in optics real time PCR tubes and 5 μL of the solution of peptide extract (50 and 100 μg/mL) and 5 μL of 100 μM SYTOX Green were added to the wells, and then the tubes were placed in a thermocycler (Agilent Mx3000p qPCR System, Agilent Technologies, Santa Clara USA). The thermocycler program was performed using the SYBR green filter selected, 40 cycles of 30 s at 37 °C (*E. coli*) or 24 °C (*A. salmonicida*), with reading at the end of each cycle. Control experiments were performed under the same conditions without the addition of peptide. Independent experiments were repeated a minimum of three times.

Deleted: 61
Deleted: company, city, country

3.5. Scanning Electron Microscopy (SEM)

A. salmonicida was cultured in TSB medium to the mid-log phase and was harvested by centrifugation at 1000× *g* for 5 min. Cell pellets were washed twice with 10 mM PBS and resuspended to an OD_{600} nm of 0.3. The cell suspension was incubated at 37 °C for 30 min with 50 μg/mL of peptide extract or phospholipase-A2-derived synthetic peptide at 20 μM. After incubation, the cells were washed three times with PBS. Bacterial pellets were then fixed overnight in 500 μL of 2.5% (*v*/*v*) glutaraldehyde in PBS at 4 °C. Subsequently, the bacterial samples were dehydrated with graded ethanol series and then dried. A small amount of platinum was sputtered on the samples to avoid charging in the microscope. Cells were examined under a scanning electron microscope (Hitachi SU 3500, Tokyo, Japan).

Comment [c37]: It is right.
Comment [M38]: Newly added information, please confirm.

3.6. Polyphenol Quantification

The content of total phenols of *S. nigra* extracts was measured by Folin-Ciocalteu spectrophotometry assay, according to the technique of Singleton and Rossi (1965) [60]. Methanol solutions of gallic acid at concentrations ranging between 100 and 1000 μg/mL were used as calibration curve. 5 μL of each gallic acid solutions and *S. nigra* extracts were used for the quantification of phenols. 75 μL of distilled water and 20 μL of 1 N Folin-Coicalteu reagent were added to each. After 3 min, 30 μL of Na_2CO_3 solution (10% *w*/*v*) was added and then 120 μL with distilled water were added. After 2 h of dark incubation, the absorbance at 760 nm was measured in a microplate reader. The content of total phenols was expressed as mg/mL.

Comment [c39]: This is not an instrument.
Deleted: (company, city, country)
Deleted: 2

4. Conclusions

AMPs are emerging as therapeutic alternatives to conventional antibiotics. Medicinal plant extracts are a plentiful source of novel agents and could be exploited in different fields, such as in animal farming infections control. The presence of cysteine-rich peptides in flowers of *S. nigra* has been determined that exhibit activity against different fish pathogens of interest in Chilean aquaculture. It is postulated that their antimicrobial activity is related to the disruption of the bacterial cell membrane. However, further studies are needed to characterize the structure and activity of each peptide present in the flowers of *S. nigra*.

Acknowledgments: This work was supported by the Chilean Fondo Nacional de Desarrollo Científico y Tecnológico (FONDECYT) Postdoctoral Grant 3160211

Author Contributions: C.A.A. and F.G. conceived of and designed the experiments and reviewed the manuscript. C.A.A harvested plant material and performed the experiments and wrote the manuscript. F.A. analyzed the data and reviewed the manuscript. M.S.R. contributed the laboratory facilities for SEM analysis. A.B. contributed the laboratory facilities for peptide characterization, performed and interpreted the LC/MS/MS analyses and reviewed the manuscript.

Conflicts of Interest: The authors declare no conflict of interest.

Comment [M40]: Please add Fanny Guzmán.

Comment [c41]: This mistake was corrected.

Deleted: A

References

Comment [M42]: 1. Ref 10 is same as ref 17, please confirm.

2. Please confirm the highlighted names in ref 13 and ref 35.

3. Ref 39 is same as ref 45, please confirm.

Comment [c43]: The references were corrected in the text.

Comment [c44]: The author names of references 13 and 34 are correct.

1. Sofowora, A.; Ogunbodede, E.; Onayade, A. The role and place of medicinal plants in the strategies for disease prevention. *Afr. J. Tradit. Complement. Altern. Med.* **2013**, *10*, 210–229.
2. Mahady, G.B. Global harmonization of herbal health claims. *J. Nutr.* **2001**, *131*, 1120S–1123S.
3. Sidor, A.; Gramza-Michałowska, A. Advanced research on the antioxidant and health benefit of elderberry (*Sambucus nigra*) in food—A review. *J. Funct. Foods* **2015**, *18*, 941–958, doi:10.1016/j.jff.2014.07.012.
4. Ho, G.T.T.; Wangensteen, H.; Barsett, H. Elderberry and elderflower extracts, phenolic compounds, and metabolites and their effect on complement, RAW 264.7 macrophages and dendritic cells. *Int. J. Mol. Sci.* **2017**, *18*, 584, doi:10.3390/ijms18030584.
5. Jabbari, M.; Daneshfard, B.; Emtiazy, M.; Khiveh, A.; Hashempur, M.H. Biological effects and clinical applications of dwarf elder (*Sambucus ebulus* L): A review. *J. Evid. Based Complement. Altern. Med.* **2017**, *22*, 996–1001, doi:10.1177/2156587217701322.
6. Krawitz, C.; Mraheil, M.A.; Stein, M.; Imirzalioglu, C.; Domann, E.; Pleschka, S.; Hain, T. Inhibitory activity of a standardized elderberry liquid extract against clinically-relevant human respiratory bacterial pathogens and influenza A and B viruses. *BMC Complement. Altern. Med.* **2011**, *11*, 16, doi:10.1186/1472-6882-11-16.
7. Nawrot, R.; Barylski, J.; Nowicki, G.; Broniarczyk, J.; Buchwald, W.; Goździcka-Józefiak, A. Plant antimicrobial peptides. *Folia Microbiol. (Praha)* **2014**, *59*, 181–196, doi:10.1007/s12223-013-0280-4.
8. Tam, J.P.; Wang, S.; Wong, K.H.; Tan, W.L. Antimicrobial peptides from plants. *Pharmaceuticals (Basel)* **2015**, *8*, 711–757, doi:10.3390/ph8040711.
9. Daly, N.L.; Rosengren, K.J.; Craik, D.J. Discovery, structure and biological activities of cyclotides. *Adv. Drug Deliv. Rev.* **2009**, *61*, 918–930, doi:10.1016/j.addr.2009.05.003.
10. Ireland, D.C.; Clark, R.J.; Daly, N.L.; Craik, D.J. Isolation, sequencing, and structure–activity relationships of cyclotides. *J. Nat. Prod.* **2010**, *73*, 1610–1622, doi:10.1021/np1000413.
11. Jennings, C.; West, J.; Waine, C.; Craik, D.; Anderson, M. Biosynthesis and insecticidal properties of plant cyclotides: The cyclic knotted proteins from *Oldenlandia affinis*. *Proc. Natl. Acad. Sci. USA* **2001**, *98*, 10614–10619, doi:10.1073/pnas.191366898.
12. Hintz, T.; Matthews, K.K.; Di, R. The use of plant antimicrobial compounds for food preservation. *Biomed Res. Int.* **2015**, *2015*, 246264, doi:10.1155/2015/246264.
13. Tavares, L.S.; Santos Mde, O.; Viccini, L.F.; Moreira, J.S.; Miller, R.N.G.; Franco, O.L. Biotechnological potential of antimicrobial peptides from flowers. *Peptides* **2008**, *29*, 1842–1851, doi:10.1016/j.peptides.2008.06.003.
14. Estomba, D.; Ladio, A.; Lozada, M. Medicinal wild plant knowledge and gathering patterns in a mapuche community from north-western patagonia. *J. Ethnopharmacol.* **2006**, *103*, 109–119, doi:10.1016/j.jep.2005.07.015.

Formatted: Not Highlight

Formatted: Highlight

Formatted: Not Highlight

15. Guerrero, C.J.; Ciampi, P.L.; Castilla, C.A.; Medel, S.F.; Schalchli, S.H.; Hormazabal, U.E.; Bensch, T.E.; Alberdi, L.M. Antioxidant capacity, anthocyanins, and total phenols of wild and cultivated berries in Chile. *Chil. J. Agric. Res.* **2010**, *70*, 537–544, doi:10.4067/S0718-58392010000400002.
16. Taneja, S.C.; Qazi, G.N. Bioactive Molecules in medicinal plants: A perspective on their therapeutic action. In *Drug Discovery and Development*; John Wiley & Sons, Inc.: Hoboken, NJ, USA, 2006; pp. 1–50, ISBN 9780470085226.
17. Ciocoiu, M.; Mirón, A.; Mares, L.; Tutunaru, D.; Pohaci, C.; Groza, M.; Badescu, M. The effects of *Sambucus nigra* polyphenols on oxidative stress and metabolic disorders in experimental diabetes mellitus. *J. Physiol. Biochem.* **2009**, *65*, 297–304, doi:10.1007/BF03180582.
18. Salvador, Â.C.; Silvestre, A.J.D.; Rocha, S.M. Unveiling elderflowers (*Sambucus nigra* L.) volatile terpenic and norisoprenoids profile: Effects of different postharvest conditions. *Food Chem.* **2017**, *229*, 276–285, doi:10.1016/j.foodchem.2017.02.037.
19. Schmitzer, V.; Veberic, R.; Slatnar, A.; Stampar, F. Elderberry (*Sambucus nigra* L.) wine: A product rich in health promoting compounds. *J. Agric. Food Chem.* **2010**, *58*, 10143–10146, doi:10.1021/jf102083s.
20. Veberic, R.; Jakopic, J.; Stampar, F.; Schmitzer, V. European elderberry (*Sambucus nigra* L.) rich in sugars, organic acids, anthocyanins and selected polyphenols. *Food Chem.* **2009**, *114*, 511–515, doi:10.1016/j.foodchem.2008.09.080.
21. Claeson, P.; Göransson, U.; Johansson, S.; Luijendijk, T.; Bohlin, L. Fractionation protocol for the isolation of polypeptides from plant biomass. *J. Nat. Prod.* **1998**, *61*, 77–81, doi:10.1021/np970342r.
22. Nguyen, G.K.T.; Zhang, S.; Wang, W.; Wong, C.T.T.; Nguyen, N.T.K.; Tam, J.P. Discovery of a linear cyclotide from the bracelet subfamily and its disulfide mapping by top-down mass spectrometry. *J. Biol. Chem.* **2011**, *286*, 44833–44844, doi:10.1074/jbc.M111.290296.
23. Nguyen, G.K.T.; Lian, Y.; Pang, E.W.H.; Nguyen, P.Q.T.; Tran, T.D.; Tam, J.P. Discovery of linear cyclotides in monocot plant *Panicum laxum* of Poaceae family provides new insights into evolution and distribution of cyclotides in plants. *J. Biol. Chem.* **2013**, *288*, 3370–3380, doi:10.1074/jbc.M112.415356.
24. Narayani, M.; Chadha, A.; Srivastava, S. Cyclotides from the indian medicinal plant *Viola odorata* (Banafsha): Identification and characterization. *J. Nat. Prod.* **2017**, *80*, 1972–1980, doi:10.1021/acs.jnatprod.6b01004.
25. Poth, A.G.; Colgrave, M.L.; Philip, R.; Kerenga, B.; Daly, N.L.; Anderson, M.A.; Craik, D.J. Discovery of cyclotides in the fabaceae plant family provides new insights into the cyclization, evolution, and distribution of circular proteins. *ACS Chem. Biol.* **2011**, *6*, 345–355, doi:10.1021/cb100388j.
26. Hashempour, H.; Koehbach, J.; Daly, N.L.; Ghassempour, A.; Gruber, C.W. Characterizing circular peptides in mixtures: Sequence fragment assembly of cyclotides from a violet plant by MALDI-TOF/TOF mass spectrometry. *Amino Acids* **2013**, *44*, 581–595, doi:10.1007/s00726-012-1376-x.
27. Koehbach, J.; Attah, A.F.; Berger, A.; Hellinger, R.; Kutchan, T.M.; Carpenter, E.J.; Rolf, M.; Sonibare, M.A.; Moody, J.O.; Wong, G.K.-S.; et al. Cyclotide discovery in Gentianales revisited-identification and characterization of cyclic cystine-knot peptides and their phylogenetic distribution in Rubiaceae plants. *Biopolymers* **2013**, *100*, 438–452, doi:10.1002/bip.22328.
28. Simonsen, S.M.; Sando, L.; Ireland, D.C.; Colgrave, M.L.; Bharathi, R.; Göransson, U.; Craik, D.J. A continent of plant defense peptide diversity: Cyclotides in *Australian Hybanthus* (Violaceae). *Plant Cell* **2005**, *17*, 3176–3189, doi:10.1105/tpc.105.034678.
29. Kathleen, M.M.; Samuel, L.; Felecia, C.; Reagan, E.L.; Kasing, A.; Lesley, M.; Toh, S.C. Antibiotic resistance of diverse bacteria from aquaculture in Borneo. *Int. J. Microbiol.* **2016**, *2016*, 2164761, doi:10.1155/2016/2164761.
30. Petersen, A.; Andersen, J.S.; Kaewmak, T.; Somsiri, T.; Dalsgaard, A. Impact of integrated fish farming on antimicrobial resistance in a pond environment. *Appl. Environ. Microbiol.* **2002**, *68*, 6036–6042, doi:10.1128/AEM.68.12.6036-6042.2002.
31. Shah, S.Q.A.; Cabello, F.C.; L'Abée-Lund, T.M.; Tomova, A.; Godfrey, H.P.; Buschmann, A.H.; Sørum, H. Antimicrobial resistance and antimicrobial resistance genes in marine bacteria from salmon aquaculture and non-aquaculture sites. *Environ. Microbiol.* **2014**, *16*, 1310–1320, doi:10.1111/1462-2920.12421.
32. Smith, P. Antimicrobial resistance in aquaculture. *Rev. Sci. Tech.* **2008**, *27*, 243–264.
33. Schmidt, A.S.; Bruun, M.S.; Dalsgaard, I.; Pedersen, K.; Larsen, J.L. Occurrence of antimicrobial resistance in fish-pathogenic and environmental bacteria associated with four danish rainbow trout farms. *Appl. Environ. Microbiol.* **2000**, *66*, 4908–4915.

34. De la Fuente, M.; Miranda, C.D.; Jopia, P.; González-Rocha, G.; Guiliani, N.; Sossa, K.; Urrutia, H. Growth Inhibition of bacterial fish pathogens and quorum-sensing blocking by bacteria recovered from chilean salmonid farms. *J. Aquat. Anim. Health* **2015**, *27*, 112–122, doi:10.1080/08997659.2014.1001534.
35. Castro, S.B.R.; Leal, C.A.G.; Freire, F.R.; Carvalho, D.A.; Oliveira, D.F.; Figueiredo, H.C.P. Antibacterial activity of plant extracts from Brazil against fish pathogenic bacteria. *Braz. J. Microbiol.* **2008**, *39*, 756–760, doi:10.1590/S1517-83822008000400030.
36. Bulfon, C.; Volpatti, D.; Galeotti, M. In Vitro antibacterial activity of plant ethanolic extracts against fish pathogens. *J. World Aquac. Soc.* **2014**, *45*, 545–557, doi:10.1111/jwas.12151.
37. Turker, H.; Yıldırım, A.B. Screening for antibacterial activity of some Turkish plants against fish pathogens: A possible alternative in the treatment of bacterial infections. *Biotechnol. Biotechnol. Equip.* **2015**, *29*, 281–288, doi:10.1080/13102818.2015.1006445.
38. Wimley, W.C. Describing the mechanism of antimicrobial peptide action with the interfacial activity model. *ACS Chem. Biol.* **2010**, *5*, 905–917, doi:10.1021/cb1001558.
39. Lee, J.; Lee, D.G. Antimicrobial Peptides (AMPs) with dual mechanisms: Membrane disruption and apoptosis. *J. Microbiol. Biotechnol.* **2015**, *25*, 759–764.
40. Brogden, K.A. Antimicrobial peptides: Pore formers or metabolic inhibitors in bacteria? *Nat. Rev. Microbiol.* **2005**, *3*, 238–250, doi:10.1038/nrmicro1098.
41. Lee, J.-K.; Park, S.-C.; Hahm, K.-S.; Park, Y. Antimicrobial HPA3NT3 peptide analogs: Placement of aromatic rings and positive charges are key determinants for cell selectivity and mechanism of action. *Biochim. Biophys. Acta* **2013**, *1828*, 443–454, doi:10.1016/j.bbamem.2012.09.005.
42. Hartmann, M.; Berditsch, M.; Hawecker, J.; Ardakani, M.F.; Gerthsen, D.; Ulrich, A.S. Damage of the bacterial cell envelope by antimicrobial peptides gramicidin S and PGLa as revealed by transmission and scanning electron microscopy. *Antimicrob. Agents Chemother.* **2010**, *54*, 3132–3142, doi:10.1128/AAC.00124-10.
43. Wimley, W.C. Determining the effects of membrane-interacting peptides on membrane integrity. *Methods Mol. Biol.* **2015**, *1324*, 89–106, doi:10.1007/978-1-4939-2806-4_6.
44. Bourbon, C.; Bry, C.; Roggemans, C.; Soulard, C.; Thizon, C.; Garbay, B. Use of a real-time polymerase chain reaction thermocycler to study bacterial cell permeabilization by antimicrobial peptides. *Anal. Biochem.* **2008**, *381*, 279–281, doi:10.1016/j.ab.2008.07.005.
45. Murillo, L.A.; Lan, C.; Agabian, N.M.; Larios, S.; Lomonte, B. Original synthetic peptide variant against *Candida albicans*. *Revista Espanola de Quimioterapia* **2007**, *20*, 330–333.
46. Meincken, M.; Holroyd, D.L.; Rautenbach, M. Atomic force microscopy study of the effect of antimicrobial peptides on the cell envelope of *Escherichia coli*. *Antimicrob. Agents Chemother.* **2005**, *49*, 4085–4092, doi:10.1128/AAC.49.10.4085-4092.2005.
47. Hale, J.D.; Hancock, R.E. Alternative mechanisms of action of cationic antimicrobial peptides on bacteria. *Expert Rev. Anti. Infect. Ther.* **2007**, *5*, 951–959, doi:10.1586/14787210.5.6.951.
48. Shai, Y. Mode of action of membrane active antimicrobial peptides. *Biopolymers* **2002**, *66*, 236–248, doi:10.1002/bip.10260.
49. Huang, H.W.; Charron, N.E. Understanding membrane-active antimicrobial peptides. *Q. Rev. Biophys.* **2017**, *50*, e10. doi:10.1017/S0033583517000087.
50. Gründemann, C.; Koehbach, J.; Huber, R.; Gruber, C.W. Do plant cyclotides have potential as immunosuppressant peptides? *J. Nat. Prod.* **2012**, *75*, 167–174, doi:10.1021/np200722w.
51. Colgrave, M.L.; Poth, A.G.; Kaas, Q.; Craik, D.J. A new "era" for cyclotide sequencing. *Biopolymers* **2010**, *94*, 592–601, doi:10.1002/bip.21400.
52. Wang, C.K.L.; Kaas, Q.; Chiche, L.; Craik, D.J. CyBase: A database of cyclic protein sequences and structures, with applications in protein discovery and engineering. *Nucleic Acids Res.* **2007**, *36*, D206–D210, doi:10.1093/nar/gkm953.
53. Mulvenna, J.P.; Wang, C.; Craik, D.J. CyBase: A database of cyclic protein sequence and structure. *Nucleic Acids Res.* **2006**, *34*, D192–D194, doi:10.1093/nar/gkj005.
54. Mitta, G.; Vandenbulcke, F.; Hubert, F.; Roch, P. Mussel defensins are synthesised and processed in granulocytes then released into the plasma after bacterial challenge. *J. Cell Sci.* **1999**, *112 Pt 2*, 4233–4242.
55. Schmitt, P.; Mercado, L.; Díaz, M.; Guzmán, F.; Arenas, G.; Marshall, S.H. Characterization and functional recovery of a novel antimicrobial peptide (CECdir-CECret) from inclusion bodies after expression in *Escherichia coli*. *Peptides* **2008**, *29*, 512–519, doi:10.1016/j.peptides.2007.12.012.

Formatted: Not Highlight

Formatted: Not Highlight

56. Jofré, C.; Guzmán, F.; Cárdenas, C.; Albericio, F.; Marshall, S.H. A natural peptide and its variants derived from the processing of infectious pancreatic necrosis virus (IPNV) displaying enhanced antimicrobial activity: A novel alternative for the control of bacterial diseases. *Peptides* **2011**, *32*, 852–858, doi:10.1016/j.peptides.2011.01.026.
57. Álvarez, C.; Acosta, F.; Montero, D.; Guzmán, F.; Torres, E.; Vega, B.; Mercado, L. Synthetic hepcidin from fish: Uptake and protection against *Vibrio anguillarum* in sea bass (*Dicentrarchus labrax*). *Fish Shellfish Immunol.* **2016**, *55*, 662–670, doi:10.1016/j.fsi.2016.06.035.
58. Miller, R.A.; Walker, R.D.; Baya, A.; Clemens, K.; Coles, M.; Hawke, J.P.; Henricson, B.E.; Hsu, H.M.; Mathers, J.J.; Oaks, J.L.; et al. Antimicrobial susceptibility testing of aquatic bacteria: Quality control disk diffusion ranges for *Escherichia coli* ATCC 25922 and *Aeromonas salmonicida subsp. salmonicida* ATCC 33658 at 22 and 28 degrees C. *J. Clin. Microbiol.* **2003**, *41*, 4318–4323, doi:10.1128/jcm.41.9.4318-4323.2003.
59. Alvarez, C.A.; Guzmán, F.; Cárdenas, C.; Marshall, S.H.; Mercado, L. Antimicrobial activity of trout hepcidin. *Fish Shellfish Immunol.* **2014**, *41*, 93–101, doi:10.1016/j.fsi.2014.04.013.
60. Singleton, V.L.; Rossi, J.A. Colorimetry of total phenolics with phosphomolybdic-phosphotungstic acid reagents. *Am. J. Enol. Vitic.* **1965**, *16*, 144–158.

Sample Availability: Samples of the compounds reported in this paper are available from the authors.

© 2018 by the authors. Submitted for possible open access publication under the terms and conditions of the Creative Commons Attribution (CC BY) license (http://creativecommons.org/licenses/by/4.0/).

Comment [M45]: Please complete this part or write not available.

Deleted: <#>Sofowora, A.; Ogunbodede, E.; Onayade, A. The role and place of medicinal plants in the strategies for disease prevention. *Afr. J. Tradit. Complement. Altern. Med.* **2013**, *10*, 210–229. ... [3]

Deleted: ......

Page 1: [1] Deleted	claudio	4/26/18 12:10:00 PM

[8] Departamento de Acuicultura, Facultad de Ciencias del Mar, Universidad Católica del Norte, Coquimbo, Chile; msromero@ucn.cl

Page 1: [2] Commented	MDPI	4/26/18 1:25:00 PM

Change hyphen to ; please confirm.

Resp: This is correct.

Page 12: [3] Deleted	claudio	4/26/18 1:45:00 PM

Sofowora, A.; Ogunbodede, E.; Onayade, A. The role and place of medicinal plants in the strategies for disease prevention. *Afr. J. Tradit. Complement. Altern. Med.* **2013**, *10*, 210–229.

Mahady, G.B. Global harmonization of herbal health claims. *J. Nutr.* **2001**, *131*, 1120S–1123S.

Sidor, A.; Gramza-Michałowska, A. Advanced research on the antioxidant and health benefit of elderberry (*Sambucus nigra*) in food—A review. *J. Funct. Foods* **2015**, *18*, 941–958, doi:10.1016/j.jff.2014.07.012.

Ho, G.T.T.; Wangensteen, H.; Barsett, H. Elderberry and elderflower extracts, phenolic compounds, and metabolites and their effect on complement, RAW 264.7 macrophages and dendritic cells. *Int. J. Mol. Sci.* **2017**, *18*, 584, doi:10.3390/ijms18030584.

Jabbari, M.; Daneshfard, B.; Emtiazy, M.; Khiveh, A.; Hashempur, M.H. Biological effects and clinical applications of dwarf elder (*Sambucus ebulus* L): A review. *J. Evid. Based Complement. Altern. Med.* **2017**, *22*, 996–1001, doi:10.1177/2156587217701322.

Krawitz, C.; Mraheil, M.A.; Stein, M.; Imirzalioglu, C.; Domann, E.; Pleschka, S.; Hain, T. Inhibitory activity of a standardized elderberry liquid extract against clinically-relevant human respiratory bacterial pathogens and influenza A and B viruses. *BMC Complement. Altern. Med.* **2011**, *11*, 16, doi:10.1186/1472-6882-11-16.

Nawrot, R.; Barylski, J.; Nowicki, G.; Broniarczyk, J.; Buchwald, W.; Goździcka-Józefiak, A. Plant antimicrobial peptides. *Folia Microbiol. (Praha)* **2014**, *59*, 181–196, doi:10.1007/s12223-013-0280-4.

Tam, J.P.; Wang, S.; Wong, K.H.; Tan, W.L. Antimicrobial peptides from plants. *Pharmaceuticals (Basel)* **2015**, *8*, 711–757, doi:10.3390/ph8040711.

Daly, N.L.; Rosengren, K.J.; Craik, D.J. Discovery, structure and biological activities of cyclotides. *Adv. Drug Deliv. Rev.* **2009**, *61*, 918–930, doi:10.1016/j.addr.2009.05.003.

Ireland, D.C.; Clark, R.J.; Daly, N.L.; Craik, D.J. Isolation, sequencing, and structure−activity relationships of cyclotides. *J. Nat. Prod.* **2010**, *73*, 1610–1622, doi:10.1021/np1000413.

Jennings, C.; West, J.; Waine, C.; Craik, D.; Anderson, M. Biosynthesis and insecticidal properties of plant cyclotides: The cyclic knotted proteins from *Oldenlandia affinis*. *Proc. Natl. Acad. Sci. USA* **2001**, *98*, 10614–10619, doi:10.1073/pnas.191366898.

Hintz, T.; Matthews, K.K.; Di, R. The use of plant antimicrobial compounds for food preservation. *Biomed Res. Int.* **2015**, *2015*, 246264, doi:10.1155/2015/246264.

Tavares, L.S.; Santos Mde, O.; Viccini, L.F.; Moreira, J.S.; Miller, R.N.G.; Franco, O.L. Biotechnological potential of antimicrobial peptides from flowers. *Peptides* **2008**, *29*, 1842–1851, doi:10.1016/j.peptides.2008.06.003.

Estomba, D.; Ladio, A.; Lozada, M. Medicinal wild plant knowledge and gathering patterns in a mapuche community from north-western patagonia. *J. Ethnopharmacol.* **2006**, *103*, 109–119, doi:10.1016/j.jep.2005.07.015.

Guerrero, C.J.; Ciampi, P.L.; Castilla, C.A.; Medel, S.F.; Schalchli, S.H.; Hormazabal, U.E.; Bensch, T.E.; Alberdi, L.M. Antioxidant capacity, anthocyanins, and total phenols of wild and cultivated berries in Chile. *Chil. J. Agric. Res.* **2010**, *70*, 537–544, doi:10.4067/S0718-58392010000400002.

Taneja, S.C.; Qazi, G.N. Bioactive Molecules in medicinal plants: A perspective on their therapeutic action. In *Drug Discovery and Development*; John Wiley & Sons, Inc.: Hoboken, NJ, USA, 2006; pp. 1–50, ISBN 9780470085226.

Ireland, D.C.; Clark, R.J.; Daly, N.L.; Craik, D.J. Isolation, sequencing, and structure-activity relationships of cyclotides. *J. Nat. Prod.* **2010**, *73*, 1610–1622, doi:10.1021/np1000413.

Ciocoiu, M.; Mirón, A.; Mares, L.; Tutunaru, D.; Pohaci, C.; Groza, M.; Badescu, M. The effects of *Sambucus nigra* polyphenols on oxidative stress and metabolic disorders in experimental diabetes mellitus. *J. Physiol. Biochem.* **2009**, *65*, 297–304, doi:10.1007/BF03180582.

Salvador, Â.C.; Silvestre, A.J.D.; Rocha, S.M. Unveiling elderflowers (*Sambucus nigra* L.) volatile terpenic and norisoprenoids profile: Effects of different postharvest conditions. *Food Chem.* **2017**, *229*, 276–285, doi:10.1016/j.foodchem.2017.02.037.

Schmitzer, V.; Veberic, R.; Slatnar, A.; Stampar, F. Elderberry (*Sambucus nigra* L.) wine: A product rich in health promoting compounds. *J. Agric. Food Chem.* **2010**, *58*, 10143–10146, doi:10.1021/jf102083s.

Veberic, R.; Jakopic, J.; Stampar, F.; Schmitzer, V. European elderberry (*Sambucus nigra* L.) rich in sugars, organic acids, anthocyanins and selected polyphenols. *Food Chem.* **2009**, *114*, 511–515, doi:10.1016/j.foodchem.2008.09.080.

Claeson, P.; Göransson, U.; Johansson, S.; Luijendijk, T.; Bohlin, L. Fractionation protocol for the isolation of polypeptides from plant biomass. *J. Nat. Prod.* **1998**, *61*, 77–81, doi:10.1021/np970342r.

Nguyen, G.K.T.; Zhang, S.; Wang, W.; Wong, C.T.T.; Nguyen, N.T.K.; Tam, J.P. Discovery of a linear cyclotide from the bracelet subfamily and its disulfide mapping by top-down mass spectrometry. *J. Biol. Chem.* **2011**, *286*, 44833–44844, doi:10.1074/jbc.M111.290296.

Nguyen, G.K.T.; Lian, Y.; Pang, E.W.H.; Nguyen, P.Q.T.; Tran, T.D.; Tam, J.P. Discovery of linear cyclotides in monocot plant *Panicum laxum* of Poaceae family provides new insights into evolution and distribution of cyclotides in plants. *J. Biol. Chem.* **2013**, *288*, 3370–3380, doi:10.1074/jbc.M112.415356.

Narayani, M.; Chadha, A.; Srivastava, S. Cyclotides from the indian medicinal plant *Viola odorata* (Banafsha): Identification and characterization. *J. Nat. Prod.* **2017**, *80*, 1972–1980, doi:10.1021/acs.jnatprod.6b01004.

Poth, A.G.; Colgrave, M.L.; Philip, R.; Kerenga, B.; Daly, N.L.; Anderson, M.A.; Craik, D.J. Discovery of cyclotides in the fabaceae plant family provides new insights into the cyclization, evolution, and distribution of circular proteins. *ACS Chem. Biol.* **2011**, *6*, 345–355, doi:10.1021/cb100388j.

Hashempour, H.; Koehbach, J.; Daly, N.L.; Ghassempour, A.; Gruber, C.W. Characterizing circular peptides in mixtures: Sequence fragment assembly of cyclotides from a violet plant by MALDI-TOF/TOF mass spectrometry. *Amino Acids* **2013**, *44*, 581–595, doi:10.1007/s00726-012-1376-x.

Koehbach, J.; Attah, A.F.; Berger, A.; Hellinger, R.; Kutchan, T.M.; Carpenter, E.J.; Rolf, M.; Sonibare, M.A.; Moody, J.O.; Wong, G.K.-S.; et al. Cyclotide discovery in Gentianales revisited-identification and characterization of cyclic cystine-knot peptides and their phylogenetic distribution in Rubiaceae plants. *Biopolymers* **2013**, *100*, 438–452, doi:10.1002/bip.22328.

Simonsen, S.M.; Sando, L.; Ireland, D.C.; Colgrave, M.L.; Bharathi, R.; Göransson, U.; Craik, D.J. A continent of plant defense peptide diversity: Cyclotides in *Australian Hybanthus* (Violaceae). *Plant Cell* **2005**, *17*, 3176–3189, doi:10.1105/tpc.105.034678.

Kathleen, M.M.; Samuel, L.; Felecia, C.; Reagan, E.L.; Kasing, A.; Lesley, M.; Toh, S.C. Antibiotic resistance of diverse bacteria from aquaculture in Borneo. *Int. J. Microbiol.* **2016**, *2016*, 2164761, doi:10.1155/2016/2164761.

Petersen, A.; Andersen, J.S.; Kaewmak, T.; Somsiri, T.; Dalsgaard, A. Impact of integrated fish farming on antimicrobial resistance in a pond environment. *Appl. Environ. Microbiol.* **2002**, *68*, 6036–6042, doi:10.1128/AEM.68.12.6036-6042.2002.

Shah, S.Q.A.; Cabello, F.C.; L'Abée-Lund, T.M.; Tomova, A.; Godfrey, H.P.; Buschmann, A.H.; Sørum, H. Antimicrobial resistance and antimicrobial resistance genes in marine bacteria from salmon aquaculture and non-aquaculture sites. *Environ. Microbiol.* **2014**, *16*, 1310–1320, doi:10.1111/1462-2920.12421.

Smith, P. Antimicrobial resistance in aquaculture. *Rev. Sci. Tech.* **2008**, *27*, 243–264.

Schmidt, A.S.; Bruun, M.S.; Dalsgaard, I.; Pedersen, K.; Larsen, J.L. Occurrence of antimicrobial resistance in fish-pathogenic and environmental bacteria associated with four danish rainbow trout farms. *Appl. Environ. Microbiol.* **2000**, *66*, 4908–4915.

de la Fuente, M.; Miranda, C.D.; Jopia, P.; González-Rocha, G.; Guiliani, N.; Sossa, K.; Urrutia, H. Growth Inhibition of bacterial fish pathogens and quorum-sensing blocking by bacteria recovered from chilean salmonid farms. *J. Aquat. Anim. Health* **2015**, *27*, 112–122, doi:10.1080/08997659.2014.1001534.

Castro, S.B.R.; Leal, C.A.G.; Freire, F.R.; Carvalho, D.A.; Oliveira, D.F.; Figueiredo, H.C.P. Antibacterial activity of plant extracts from Brazil against fish pathogenic bacteria. *Braz. J. Microbiol.* **2008**, *39*, 756–760, doi:10.1590/S1517-83822008000400030.

Bulfon, C.; Volpatti, D.; Galeotti, M. In Vitro antibacterial activity of plant ethanolic extracts against fish pathogens. *J. World Aquac. Soc.* **2014**, *45*, 545–557, doi:10.1111/jwas.12151.

Turker, H.; Yıldırım, A.B. Screening for antibacterial activity of some Turkish plants against fish pathogens: A possible alternative in the treatment of bacterial infections. *Biotechnol. Biotechnol. Equip.* **2015**, *29*, 281–288, doi:10.1080/13102818.2015.1006445.

Wimley, W.C. Describing the mechanism of antimicrobial peptide action with the interfacial activity model. *ACS Chem. Biol.* **2010**, *5*, 905–917, doi:10.1021/cb1001558.

Lee, J.; Lee, D.G. Antimicrobial Peptides (AMPs) with dual mechanisms: Membrane disruption and apoptosis. *J. Microbiol. Biotechnol.* **2015**, *25*, 759–764.

Brogden, K.A. Antimicrobial peptides: Pore formers or metabolic inhibitors in bacteria? *Nat. Rev. Microbiol.* **2005**, *3*, 238–250, doi:10.1038/nrmicro1098.

Lee, J.-K.; Park, S.-C.; Hahm, K.-S.; Park, Y. Antimicrobial HPA3NT3 peptide analogs: Placement of aromatic rings and positive charges are key determinants for cell selectivity and mechanism of action. *Biochim. Biophys. Acta* **2013**, *1828*, 443–454, doi:10.1016/j.bbamem.2012.09.005.

Hartmann, M.; Berditsch, M.; Hawecker, J.; Ardakani, M.F.; Gerthsen, D.; Ulrich, A.S. Damage of the bacterial cell envelope by antimicrobial peptides gramicidin S and PGLa as revealed by transmission and scanning electron microscopy. *Antimicrob. Agents Chemother.* **2010**, *54*, 3132–3142, doi:10.1128/AAC.00124-10.

Wimley, W.C. Determining the effects of membrane-interacting peptides on membrane integrity. *Methods Mol. Biol.* **2015**, *1324*, 89–106, doi:10.1007/978-1-4939-2806-4_6.

William, W.C. Describing the mechanism of antimicrobial peptide action with the interfacial activity Model. *ACS Chem. Biol.* **2010**, *5*, 905–917.doi:10.1021/cb1001558.

Bourbon, C.; Bry, C.; Roggemans, C.; Soulard, C.; Thizon, C.; Garbay, B. Use of a real-time polymerase chain reaction thermocycler to study bacterial cell permeabilization by antimicrobial peptides. *Anal. Biochem.* **2008**, *381*, 279–281, doi:10.1016/j.ab.2008.07.005.

Murillo, L.A.; Lan, C.; Agabian, N.M.; Larios, S.; Lomonte, B. Original synthetic peptide variant against *Candida albicans*. *Revista Espanola de Quimioterapia* **2007**, *20*, 330–333.

Meincken, M.; Holroyd, D.L.; Rautenbach, M. Atomic force microscopy study of the effect of antimicrobial peptides on the cell envelope of *Escherichia coli*. *Antimicrob. Agents Chemother.* **2005**, *49*, 4085–4092, doi:10.1128/AAC.49.10.4085-4092.2005.

Hale, J.D.; Hancock, R.E. Alternative mechanisms of action of cationic antimicrobial peptides on bacteria. *Expert Rev. Anti. Infect. Ther.* **2007**, *5*, 951–959, doi:10.1586/14787210.5.6.951.

Shai, Y. Mode of action of membrane active antimicrobial peptides. *Biopolymers* **2002**, *66*, 236–248, doi:10.1002/bip.10260.

Huang, H.W.; Charron, N.E. Understanding membrane-active antimicrobial peptides. *Q. Rev. Biophys*. **2017**, *50*, e10. doi:10.1017/S0033583517000087.

Gründemann, C.; Koehbach, J.; Huber, R.; Gruber, C.W. Do plant cyclotides have potential as immunosuppressant peptides? *J. Nat. Prod.* **2012**, *75*, 167–174, doi:10.1021/np200722w.

Colgrave, M.L.; Poth, A.G.; Kaas, Q.; Craik, D.J. A new "era" for cyclotide sequencing. *Biopolymers* **2010**, *94*, 592–601, doi:10.1002/bip.21400.

Wang, C.K.L.; Kaas, Q.; Chiche, L.; Craik, D.J. CyBase: A database of cyclic protein sequences and structures, with applications in protein discovery and engineering. *Nucleic Acids Res.* **2007**, *36*, D206–D210, doi:10.1093/nar/gkm953.

Mulvenna, J.P.; Wang, C.; Craik, D.J. CyBase: A database of cyclic protein sequence and structure. *Nucleic Acids Res.* **2006**, *34*, D192–D194, doi:10.1093/nar/gkj005.

Mitta, G.; Vandenbulcke, F.; Hubert, F.; Roch, P. Mussel defensins are synthesised and processed in granulocytes then released into the plasma after bacterial challenge. *J. Cell Sci.* **1999**, *112 Pt 2*, 4233–4242.

Schmitt, P.; Mercado, L.; Díaz, M.; Guzmán, F.; Arenas, G.; Marshall, S.H. Characterization and functional recovery of a novel antimicrobial peptide (CECdir-CECret) from inclusion bodies after expression in *Escherichia coli*. *Peptides* **2008**, *29*, 512–519, doi:10.1016/j.peptides.2007.12.012.

Jofré, C.; Guzmán, F.; Cárdenas, C.; Albericio, F.; Marshall, S.H. A natural peptide and its variants derived from the processing of infectious pancreatic necrosis virus (IPNV) displaying enhanced antimicrobial activity: A novel alternative for the control of bacterial diseases. *Peptides* **2011**, *32*, 852–858, doi:10.1016/j.peptides.2011.01.026.

Álvarez, C.; Acosta, F.; Montero, D.; Guzmán, F.; Torres, E.; Vega, B.; Mercado, L. Synthetic hepcidin from fish: Uptake and protection against *Vibrio anguillarum* in sea bass (*Dicentrarchus labrax*). *Fish Shellfish Immunol.* **2016**, *55*, 662–670, doi:10.1016/j.fsi.2016.06.035.
Miller, R.A.; Walker, R.D.; Baya, A.; Clemens, K.; Coles, M.; Hawke, J.P.; Henricson, B.E.; Hsu, H.M.; Mathers, J.J.; Oaks, J.L.; et al. Antimicrobial susceptibility testing of aquatic bacteria: Quality control disk diffusion ranges for *Escherichia coli* ATCC 25922 and *Aeromonas salmonicida subsp. salmonicida* ATCC 33658 at 22 and 28 degrees C. *J. Clin. Microbiol.* **2003**, *41*, 4318–4323, doi:10.1128/jcm.41.9.4318-4323.2003.
Alvarez, C.A.; Guzmán, F.; Cárdenas, C.; Marshall, S.H.; Mercado, L. Antimicrobial activity of trout hepcidin. *Fish Shellfish Immunol.* **2014**, *41*, 93–101, doi:10.1016/j.fsi.2014.04.013.
Singleton, V.L.; Rossi, J.A. Colorimetry of total phenolics with phosphomolybdic-phosphotungstic acid reagents. *Am. J. Enol. Vitic.* **1965**, *16*, 144–158.
